# Supplementary material for: Chimeric Antigen Receptor T Cells in Glioblastoma—Current Concepts and Promising Future
Source: Cells. 2023 Jul 3;12(13):1770. doi: 10.3390/cells12131770 (PMC10340625; doi:10.3390/cells12131770)
Supplement: Supplementary file 1 [file cells-12-01770-s001.zip › cells-2426275-supplementary.pdf]

Supplementary Material

Table S1. Targets used in preclinical in clinical studies and their characteristics.

Target	Characteristics	Preclinical Studies	Clinical Studies
IL13Rα	• Present in over 75% of GBMs and is associated with increased tumor invasiveness and progression, and thus a poorer prognosis [117,118]<br>• The receptor is overexpressed in GBM but not in normal tissue [119]	[28–32,41,103]	[57–59]
EGFRvIII	• Transmembrane protein which influences the migration of neuronal stem cells during development and promotes cellular proliferation [120]<br>• Wild-type EGFR is not detected in normal brain tissue, but is amplified in about 40% of GBM [120]<br>• The EGFR gene exhibits various mutations, e.g., the EGFRvIII, which can be found in about 50% of GBM patients [5,19]	[33–35,37–40,97,103,107,108,121,122]	[60,61]
HER2	• Receptor tyrosine kinase, which is overexpressed in many human cancers, such as breast cancer, ovarian cancer and in 76% of primary GBM lines [24,75]<br>• Not tumor specific, expressed in normal tissue [27]	[30,31]	[62]
EphA2	• Strongly overexpressed in 60% of GBMs, as well as GCS and at low levels on adult proliferating epithelial cells and brain tissue [24,115]	[31,41,42]	[63]
NKG2DL	• Natural killer group, member D ligands (NKGD2L) is upregulated in infected and tumor cells, while the corresponding receptor is expressed by NK cells and CD8+ cells [123]<br>• Can be induced in non-tumoral tissue in case of unphysiological cell stress or inflammation [124,125}	[45,46]	
GD2	• Disaloganglioside, which is highly expressed in GBM [49]	[47–49]	[64,65]
B7-H3	• Immune checkpoint molecule, which binds to about 70% of neuroepithelial tumors without binding normal glia or tissue [27]	[50,51]	
CD70	• Transmembrane protein and member of the TNF-family, which is not present in normal peripheral and brain tissues, but is overexpressed in about 92% of tested cell lines in GBM and is associated with T cell dysfunction and apoptosis [24,115]	[53–55,93]	
CD133	• Marker for self-renewing GSCs [27]	[56]	
